# ALDH2 Deficiency Promotes Mitochondrial Oxidative Stress and Adverse Limb Outcomes via VDAC–HSP60 Signaling

**DOI:** 10.1002/advs.78668

**Published:** 2026-11-09

**Authors:** You‐Cheng Lin, Nan‐Chun Wu, Chon‐Seng Hong, Po‐Chao Hsu, Wen‐Jin Yang, Yi‐Cheng Chang, Che‐Hong Chen, Wei‐Ting Chang

**Affiliations:** ^1^ Division of Plastic Surgery Department of Surgery Chi‐Mei Medical Center Tainan Taiwan; ^2^ Division of Cardiovascular Surgery Department of Surgery Chi Mei Medical Center Tainan Taiwan; ^3^ Department of Hospital and Health Care Administration Chia Nan University of Pharmacy and Science Tainan Taiwan; ^4^ Division of Cardiology Department of Internal Medicine Chi Mei Medical Center Tainan Taiwan; ^5^ Department of Health and Nutrition Chia Nan University of Pharmacy and Science Tainan Taiwan; ^6^ Division of Cardiology Department of Internal Medicine Kaohsiung Medical University Hospital Kaohsiung Medical University Kaohsiung Taiwan; ^7^ Foresee Pharmaceuticals, Co. Ltd. Taipei Taiwan; ^8^ Graduate Institute of Medical Genomics and Proteomics National Taiwan University Taipei Taiwan; ^9^ Department of Internal Medicine National Taiwan University Hospital Taipei Taiwan; ^10^ Institute of Biomedical Sciences Academia Sinica Taipei Taiwan; ^11^ Department of Chemical and Systems Biology Stanford University School of Medicine Stanford California USA; ^12^ School of Medicine College of Medicine National Sun Yat‐Sen University Kaohsiung Taiwan; ^13^ School of Medicine and Doctoral Program of Clinical and Experimental Medicine College of Medicine and Center of Excellence for Metabolic Associated Fatty Liver Disease National Sun Yat‐Sen University Kaohsiung Taiwan

**Keywords:** 4‐HNE, ALDH2 deficiency, HSP60 (HSPD1), mitochondrial oxidative stress, peripheral artery disease (PAD)

## Abstract

The ALDH2 rs671 variant, which is frequent in East Asian populations, markedly reduces mitochondrial aldehyde‐detoxifying capacity and thereby increases vulnerability to reactive aldehydes, including 4‐hydroxynonenal (4‐HNE), during ischemic stress. We show that impaired ALDH2 activity perturbs endothelial mitochondrial proteostasis through an ALDH2–4‐HNE–HSP60–VDAC1 pathway. In Aldh2 knock‐in mice and endothelial cells exposed to oxygen–glucose deprivation, ALDH2 deficiency was accompanied by 4‐HNE accumulation, mitochondrial DNA injury, loss of membrane potential, increased mitochondrial ROS, mitochondrial fragmentation, and apoptosis. Analysis of transcriptomic data highlighted HSP60 (HSPD1) as a downstream candidate. LC–MS/MS, covalent docking, co‐immunoprecipitation, and proximity ligation analyses supported 4‐HNE modification of HSP60, while native electrophoresis indicated disruption of higher‐order HSP60 assembly. HSP60 redistribution to the cytosol was predominantly dependent on VDAC1. Activating ALDH2 with AD‐5591, or suppressing VDAC1 genetically or pharmacologically, preserved HSP60 homeostasis, mitochondrial integrity, and endothelial function. In a prospective cohort of 227 patients with peripheral artery disease, the rs671 variant was associated with a higher incidence of major adverse limb events. These data position HSP60 proteostasis downstream of ALDH2‐dependent aldehyde detoxification and identify the ALDH2–4‐HNE–HSP60–VDAC1 pathway as a potential therapeutic target in ischemic vascular disease.

## Graphical abstract

ALDH2 deficiency promotes 4‐HNE accumulation, resulting in covalent modification of HSP60, disruption of its oligomeric assembly, and VDAC1‐dependent release of HSP60 from mitochondria to the cytosol. Pharmacological activation of ALDH2 preserves HSP60 proteostasis and mitochondrial integrity, whereas the clinical association between the ALDH2 rs671 polymorphism and adverse limb outcomes supports the translational significance of this pathway.

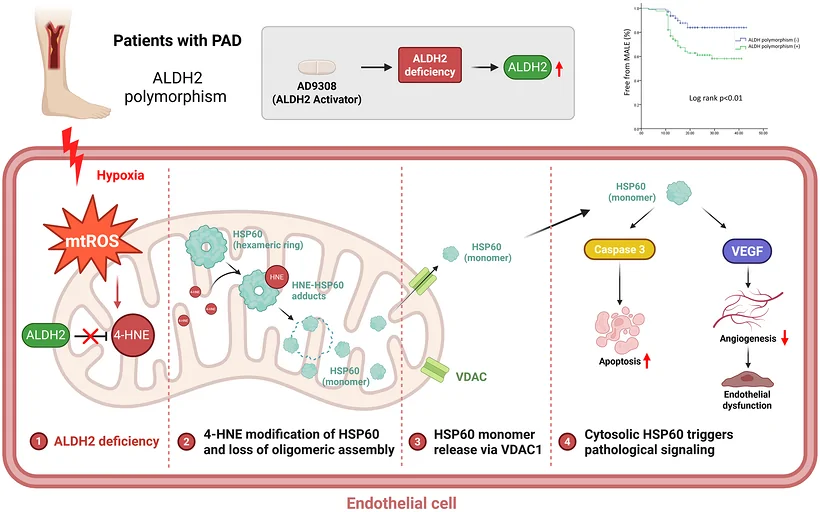

## 1 Introduction

Lower‐extremity ischemia in peripheral artery disease (PAD) reflects not only arterial obstruction but also failure of vascular adaptation [1, 2]. Oxidant generation within ischemic tissue can convert membrane lipids into reactive aldehydes, among which 4‐hydroxy‐2‐nonenal (4‐HNE) is particularly capable of modifying proteins [2, 3]. Mitochondrial aldehyde dehydrogenase 2 (ALDH2) provides an important route for eliminating these products [4, 5]. The rs671 G>A variant (Glu504Lys) markedly decreases ALDH2 activity and is common in East Asian populations, while carriers have greater aldehydic and oxidative stress and may be more vulnerable to ischemic injury [6, 7]. Experimental enhancement of ALDH2 activity reduces such injury [6, 7]. AD‐5591, a newer ALDH2 activator, has greater activating potency than Alda‐1, in part because of an additional interaction with Ala461, and has shown metabolic benefit in Aldh2 mouse models [7].

A potential consequence of aldehyde accumulation is loss of mitochondrial protein quality control. HSP60, also known as HSPD1, is a mitochondrial chaperonin that functions in multimeric complexes to maintain protein folding and organelle integrity [8, 9]. Cellular stress can alter both its assembly state and subcellular distribution [9]. VDAC proteins, located in the outer mitochondrial membrane, regulate exchange between mitochondria and cytosol and participate in stress‐associated permeability signaling [10, 11]. VDAC1 has been particularly implicated in mitochondrial destabilization [10, 11]. It remains unclear whether ALDH2‐dependent aldehydic stress directly modifies HSP60, alters its oligomeric state, or promotes VDAC‐mediated redistribution during endothelial ischemia. We addressed these questions across endothelial cells, an Aldh2 knock‐in hindlimb‐ischemia model, and a prospective PAD cohort, and additionally tested whether ALDH2 activation or VDAC inhibition could interrupt this pathway. This study investigated the role of ALDH2 in ischemia‐induced endothelial dysfunction and revascularization and delineated the underlying molecular mechanisms linking aldehydic stress to mitochondrial injury. Using endothelial cell and Aldh2‐deficient mouse models, together with a prospective clinical cohort of patients with PAD, we examined the effects of ALDH2 deficiency on mitochondrial homeostasis, angiogenic capacity, and ischemic outcomes and investigated the involvement of the 4‐HNE–HSP60–VDAC signaling axis. We further evaluated whether pharmacological activation of ALDH2 could mitigate these pathological responses.

## 2 Results

### 2.1 ALDH2 Deficiency Impairs Perfusion Recovery and Increases Oxidative Stress After Limb Ischemic Conditions

To clarify the role of ALDH2 in vascular repair and post‐ischemic angiogenesis, we established a murine hindlimb ischemia model in 10‐week‐old both wild‐type (WT) and Aldh2 knock‐in (KI) mice, a Aldh2‐deficient mouse model. Serial laser Doppler perfusion imaging revealed a marked impairment in blood flow recovery in Aldh2 KI mice compared with WT controls. While WT mice demonstrated progressive reperfusion beginning on day 14, Aldh2 KI mice exhibited persistently reduced perfusion (Figure 1A,B). Histological and immunofluorescence analyses further supported these findings. In ischemic muscle tissue, Aldh2 KI mice displayed significantly fewer CD31‐positive capillaries, indicating diminished angiogenic capacity, accompanied by enhanced accumulation of 4‐HNE, a toxic lipid peroxidation product, compared with WT mice (Figure 1C–E). Furthermore, Aldh2 KI mice showed elevated DNA damage levels, detected by 8OHdG staining, post hindlimb ischemia compared with WT mice (Figure 1F–H).

**FIGURE 1:**
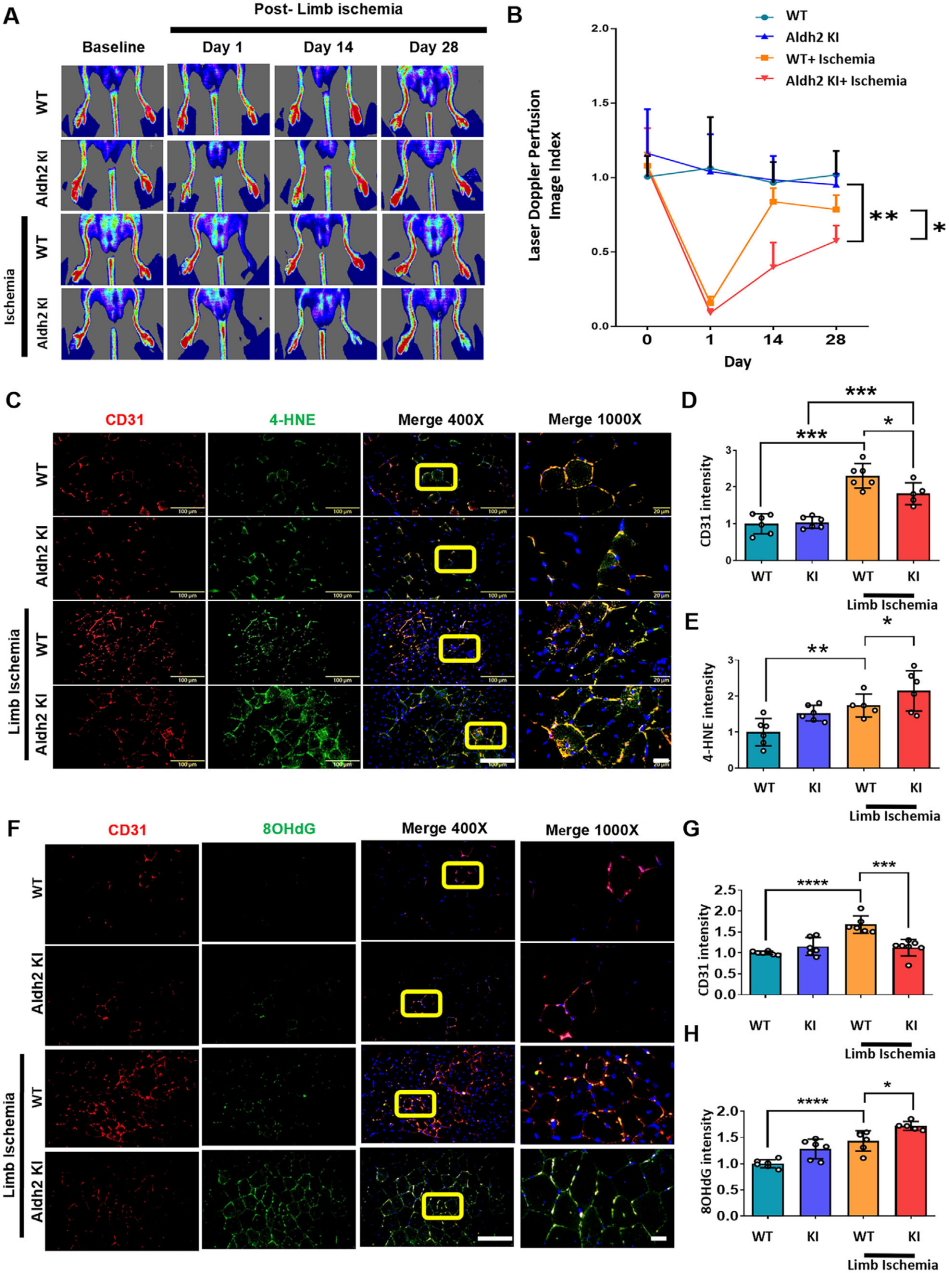
ALDH2 deficiency impairs perfusion recovery and enhances oxidative stress following limb ischemia. (A) Representative laser Doppler perfusion images of wild‐type (WT) and ALDH2 knock‐in (KI) mice at baseline (Day 0) and after hindlimb ischemia (Days 1, 14, and 28). (B) Quantitative analysis of perfusion recovery, expressed as perfusion intensity index (n = 5 per group). (C) Representative immunofluorescence staining of ischemic gastrocnemius muscle showing CD31 (red), 4‐HNE (green), and DAPI (blue). Scale bars are 100 and 20 µm at 400× and 1000× magnification, respectively. (D) Quantification of CD31 fluorescence intensity demonstrating impaired angiogenesis in ALDH2 KI mice. (E) Quantification of 4‐HNE fluorescence intensity showing elevated oxidative stress in ALDH2 KI mice. (F‐H) Schematic representation and quantification of DNA damage (8OHdG) accumulation in ischemic tissue. Data are presented as mean ± SEM. Statistical significance was determined using two‐way ANOVA followed by post hoc testing; ^*^ *p* < 0.05, ^**^ *p* < 0.01, ^***^ *p* < 0.005, ^****^ *p* < 0.001.

### 2.2 AD‐5591 Rescued ALDH2 Deficiency Associated Impairment of Endothelial Functions

AD‐5591, a new‐generation small molecule of selective ALDH2 activator, has been reported to enhance aldehyde detoxification by promoting 4‐HNE clearance [7]. However, its effect on endothelial function remains uncertain. As shown in Table S1, pharmacokinetic analysis demonstrated that AD‐5591 exhibited rapid systemic exposure following intravenous administration in male C57BL/6 mice, with a Tmax of 0.1 h and a Cmax of 2860 ng/mL. The compound showed a moderate elimination half‐life (t1/2 = 6.01 h) and systemic clearance of 3410 mL/hr/kg, indicating relatively rapid distribution and elimination. The volume of distribution (Vss = 4540 mL/kg) suggests extensive tissue distribution. Following oral administration (10 mg/kg), AD‐5591 reached peak plasma concentration at 0.5 h with a Cmax of 429 ng/mL and a shorter apparent half‐life (3.6 h). The calculated oral bioavailability was 17.6%, indicating moderate absorption.

In Figure 2A, treatment of 10 µm AD‐5591 significantly elevated ALDH2 activity in human umbilical vein endothelial cells (HUVECs). Also, BrdU incorporation assays demonstrated diminished proliferation in siALDH2 + oxygen‐glucose deprivation (OGD) cells, which was rescued by AD5591 (Figure 2B). In a cell migration assay, siALDH2‐transfected HUVECs exhibited reduced migration compared with controls, and this defect was exacerbated by OGD, while treatment with AD5591 restored migratory capacity (Figure 2C,D). Furthermore, tube formation assays showed that both siALDH2 and OGD suppressed angiogenic network formation, as indicated by decreased branch point numbers, while AD5591 treatment improved tube formation (Figure 2E,F). Also, siALDH2 and OGD treatment induced DNA damage and ROS accumulation, which were rescued by AD5591 in HUVECs (Figure G‐J). These findings suggest that ALDH2 activity is essential for maintaining endothelial migration, proliferation, and angiogenesis under ischemic‐like conditions, and pharmacological activation with AD5591 mitigates these deficits.

**FIGURE 2:**
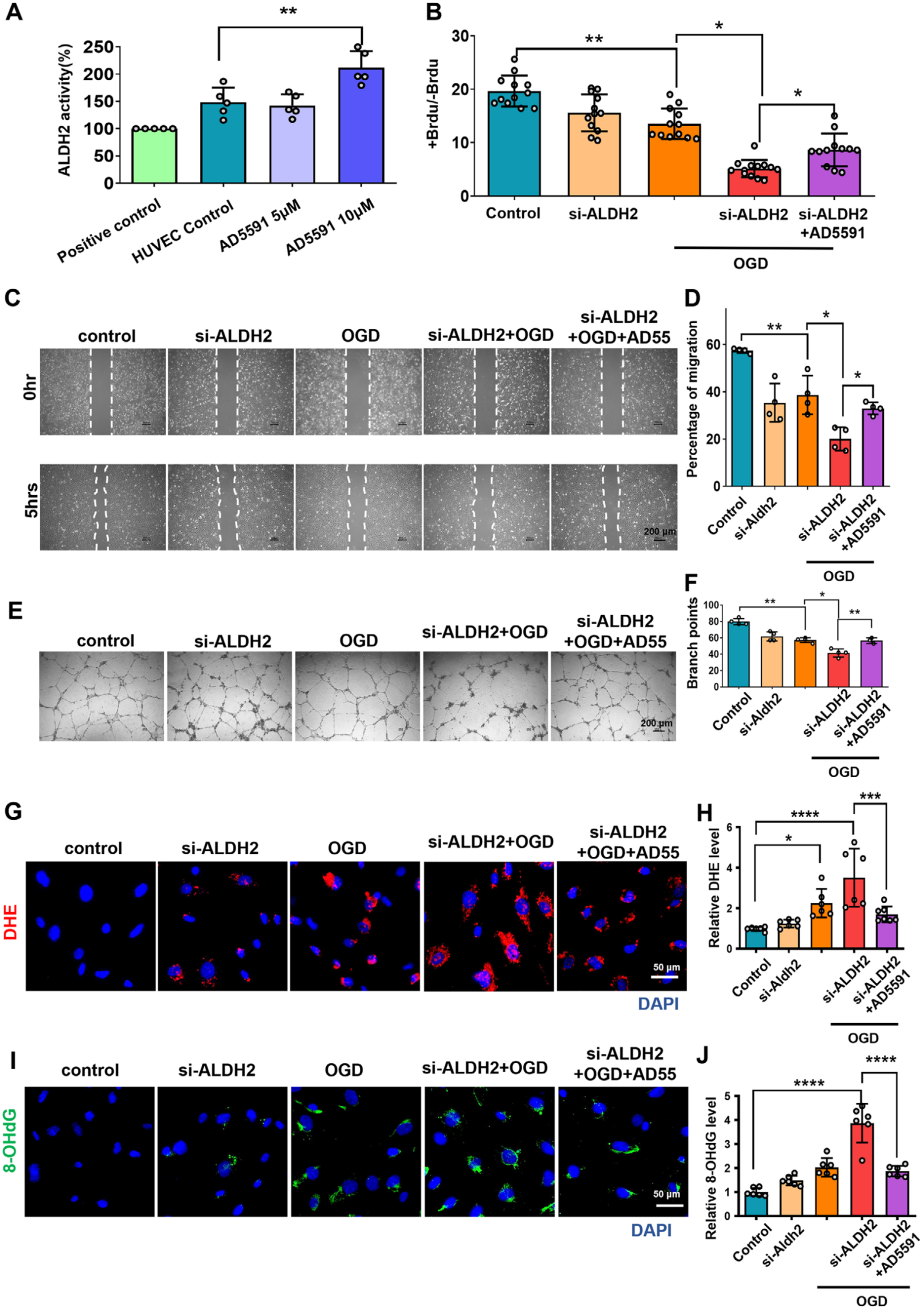
ALDH2 knockdown impairs endothelial migration, proliferation, and angiogenic capacity under hypoxic conditions, rescued by AD5591. (A) ALDH2 enzymatic activity assay showing dose‐dependent activation by AD5591 in HUVECs (n = 5 per group). (B) Endothelial proliferation assessed by BrdU incorporation. siALDH2 combined with OGD significantly reduced proliferation, which was restored by AD5591 (n = 12 per group). (C‐D) Representative images and quantification of migration assays at 0 and 5 h under the indicated conditions. Red lines indicate scratch edges (n = 4–6 per group). (E‐F) Representative tube formation images and quantification demonstrating reduced angiogenic network formation in siALDH2 and OGD groups, with AD5591 treatment improving tube formation. Representative images and quantification of (G‐H) DHE (Dihydroethidium), cellular reactive oxygen species (ROS), and (I‐J) 8OHdG, DNA damage, expression in the indicated groups. Data are presented as mean ± SEM. Statistical significance was determined using one‐way ANOVA with post hoc testing; ^*^ *p* < 0.05, ^**^ *p* < 0.01, ^***^ *p* < 0.005, ^****^ *p* < 0.001.

### 2.3 Transcriptomic Profiling Identifies HSP60 as a Downstream Target of ALDH2 Deficiency

To investigate the molecular mechanisms of endothelial dysfunction, we re‐analyzed the bulk RNA‐seq dataset GSE184419, which includes Aldh2‐corrected isogenic human induced pluripotent stem cells (hiPSC)‐derived endothelial cells (Iso_GG) and uncorrected Aldh2 1/^*^2 counterparts [12]. Functional enrichment revealed significant alterations in oxidative stress–related pathways, with Gene Ontology highlighting processes linked to apoptosis, transcriptional regulation, and inflammation (Figure 3A). Volcano plots and heatmaps emphasized DEGs such as GSTP1, APOE, CAT, and Heat Shock Protein Family D Member 1 (HSPD1) encoding HSP60, while STRING network analysis also suggested functional associations between ALDH2, HSP60, and oxidative stress modulators (Figure S1 and Figure 3B,C). HSP60, a mitochondrial chaperone that maintains protein folding and mitochondrial integrity, has been reported to mitigate oxidative stress–induced mitochondrial injury [8, 9]. We further examined the expression of HSP60 in the murine hindlimb ischemia model and observed a significant increase of HSP60 accumulation in ischemic muscle of Aldh2 KI mice compared with WT, linking transcriptomic alterations to in vivo oxidative stress responses (Figure 3D,E).

**FIGURE 3:**
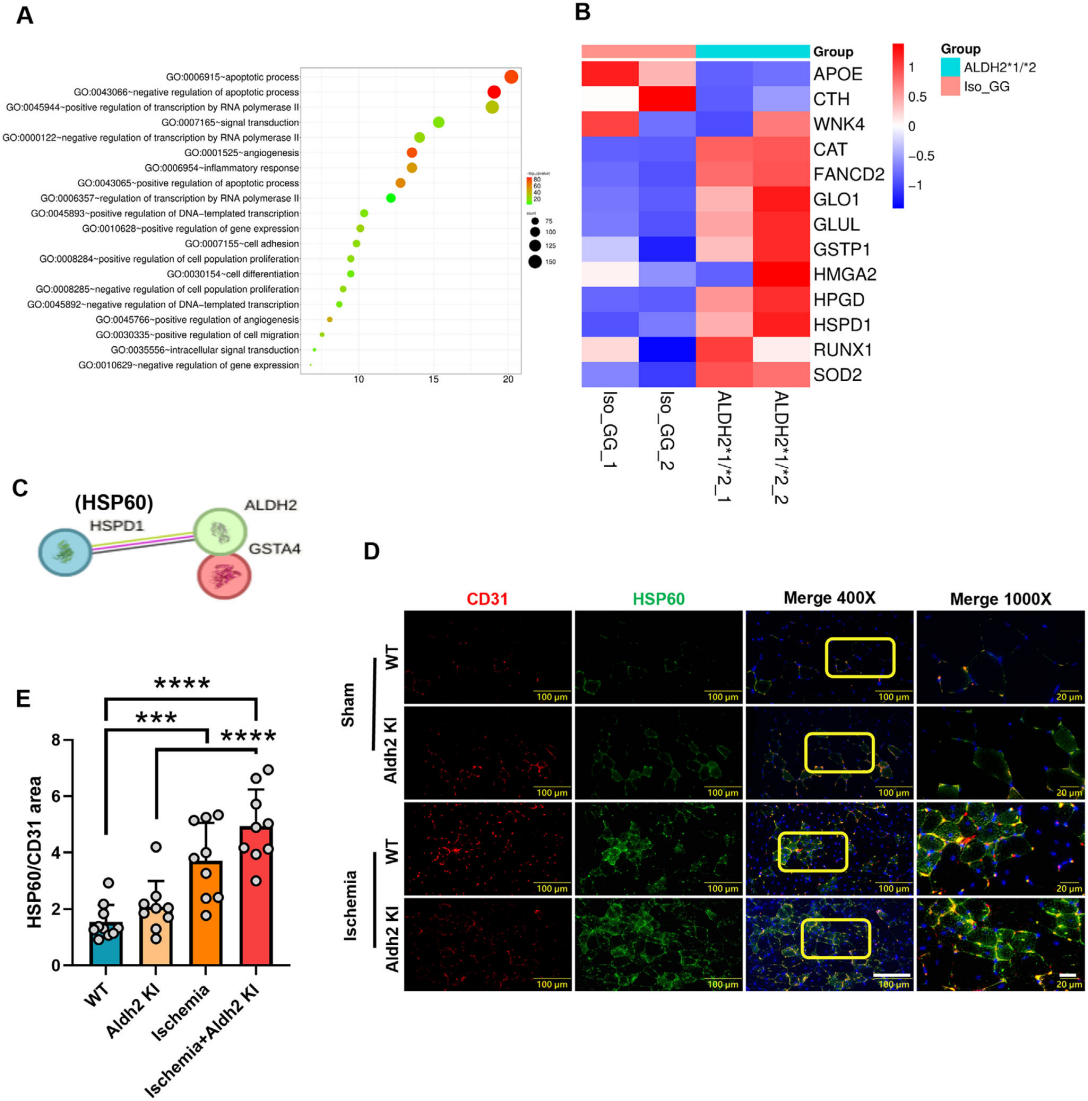
Integrated transcriptomic and experimental analyses identify HSP60 as a downstream effector of ALDH2 deficiency during ischemic stress. (A) Gene Ontology (GO) enrichment analysis of differentially expressed genes (DEGs) highlights processes related to apoptosis regulation, transcriptional control, and inflammatory response. (B) Heatmap of representative DEGs shows altered expression of oxidative stress–associated genes such as APOE, CAT, GSTP1, and HSPD1 (HSP60). (C) STRING protein–protein interaction network reveals functional connections between ALDH2 and oxidative stress–modulating proteins, notably GSTP1 and HSPD1 (HSP60). (D‐E) Representative immunofluorescence staining of CD31 (red) and HSP60 (green) in sham and ischemic muscle from WT and ALDH2 KI mice (n = 5 per group). Scale bars are 100 and 20 µm at 400× and 1000× magnification, respectively. Data are presented as mean ± SEM. Statistical significance was determined using one‐way ANOVA with post hoc testing; ^***^ *p* < 0.005, ^****^ *p* < 0.001.

### 2.4 ALDH2 Deficiency Triggered HSP60 Mislocalization, ROS and Apoptosis Which Was Rescued by ALDH2 Activation (AD5591)

ALDH2 deficiency significantly aggravated OGD‐induced intracellular 4‐HNE accumulation and promoted HSP60 redistribution from mitochondria to the cytosol, whereas treatment with the ALDH2 activator AD5591 partially reversed these effects (Figure 4A,B). To investigate whether HSP60 serves as a direct molecular target of 4‐HNE, liquid chromatography‐tandem mass spectrometry (LC‐MS/MS) analysis identified 4‐HNE covalent adduct formation on the HSP60 peptide, suggesting HSP60 as a direct molecular target of 4‐HNE‐mediated carbonyl modification. Also, covalent docking analysis predicted a favorable interaction between 4‐HNE and HSP60, identifying a potential binding region near lysine 335 (K335), where the reactive aldehyde moiety was positioned in close proximity to facilitate covalent adduct formation. In addition, 4‐HNE formed a stabilizing hydrogen bond with serine 374 (S374) within the predicted binding pocket (Figure 4C). Consistent with these structural predictions, co‐immunoprecipitation and proximity ligation assays demonstrated significantly enhanced 4‐HNE‐mediated HSP60 carbonyl modification under ALDH2 deficiency and ischemic stress, which was attenuated by AD5591 treatment (Figure 4D,E). Whole‐cell protein analysis further demonstrated reduced VEGF along with elevated cleaved caspase‐3 expression, indicative of enhanced apoptosis and impaired angiogenic signaling (Figure S2). Collectively, these findings suggest that impaired ALDH2 activity promotes 4‐HNE‐dependent covalent modification of HSP60, leading to altered subcellular redistribution of HSP60 and disruption of mitochondrial proteostasis during ischemic injury.

**FIGURE 4:**
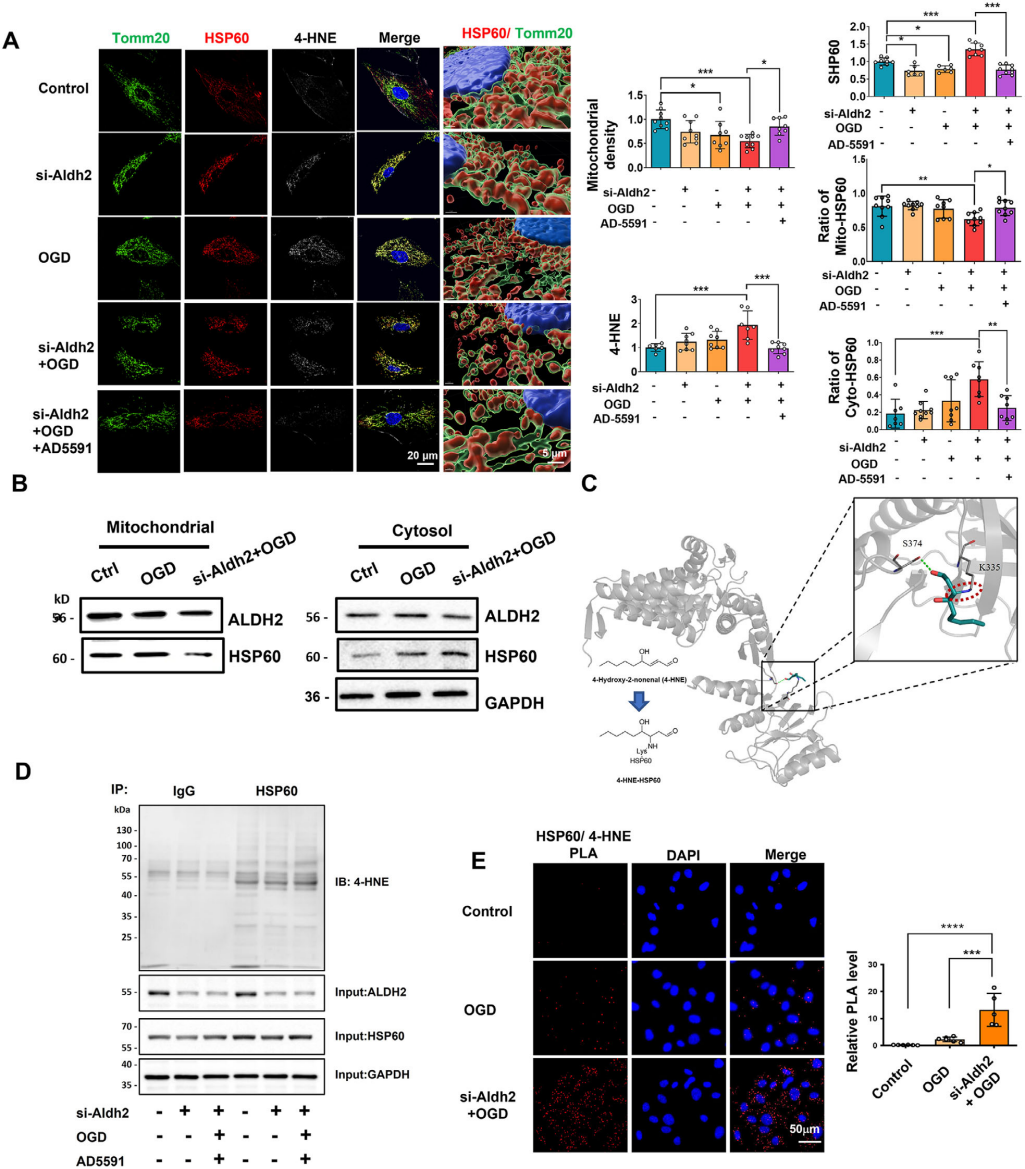
ALDH2 deficiency enhances 4‐HNE‐mediated HSP60 modification and promotes mitochondrial redistribution of HSP60 under ischemic stress. (A) Representative immunofluorescence images of HUVECs stained for mitochondrial marker TOM20 (green), HSP60 (red), and 4‐HNE (gray) under control, si‐Aldh2, OGD, si‐Aldh2 + OGD, and si‐Aldh2 + OGD + AD5591 conditions. Quantitative analysis of cytosolic HSP60 intensity, total HSP60 expression, and intracellular 4‐HNE levels is shown on the right (n = 4–6/group). (B) Representative immunoblot analysis of mitochondrial and cytosolic fractions showing redistribution of ALDH2 and HSP60 following OGD and ALDH2 silencing. GAPDH was used as a cytosolic loading control. (C) Covalent docking model of 4‐HNE with human HSP60. Covalent docking of 4‐HNE to human HSP60 was performed using SeeSAR. The overall docking pose is shown on the left, with HSP60 represented as a gray cartoon and 4‐HNE shown as cyan sticks. The enlarged view highlights the predicted binding environment of 4‐HNE near K335. The putative covalent interaction site is indicated by the red dashed circle, and the hydrogen bond is shown as a green dashed line. (D) Co‐immunoprecipitation analysis using anti‐HSP60 antibody followed by immunoblotting for 4‐HNE, demonstrating increased 4‐HNE adduct formation on HSP60 under OGD and ALDH2 knockdown conditions. AD5591 partially attenuated this interaction. (E) Proximity ligation assay (PLA) detecting direct interaction between HSP60 and 4‐HNE in HUVECs. Representative fluorescence images and quantitative analysis demonstrate significantly enhanced HSP60–4‐HNE interaction following OGD, which was further augmented by ALDH2 silencing (scale bar = 50 µm). ^*^ *p* < 0.05, ^**^ *p* < 0.01, ^***^ *p* < 0.005, ^****^ *p* < 0.001. (N = 4‐7 biologically independent samples).

### 2.5 ALDH2 Deficiency Exacerbates Mitochondrial Dysfunction Under Ischemic Stress

To investigate the mitochondrial consequences of ALDH2 deficiency under ischemic stress, we assessed mitochondrial integrity, morphology, membrane potential, and oxidative stress in HUVECs subjected to OGD. Quantitative PCR demonstrated a significant reduction in mitochondrial DNA copy number (mtDNA/β‐globin ratio) following OGD, which was further exacerbated by ALDH2 knockdown, indicating aggravated mitochondrial damage (Figure 5A). Mitochondrial reactive oxygen species (MitoSOX) red staining demonstrated excessive mitochondrial ROS accumulation in ALDH2‐deficient cells under ischemic stress (Figure 5B). Consistently, JC‐1 flow cytometric analysis showed marked mitochondrial membrane depolarization (Figure 5C). Confocal microscopy revealed pronounced mitochondrial fragmentation in the siAldh2 + OGD condition, characterized by shortened, punctate mitochondrial structures and loss of network continuity (Figure 5D–H), while Pharmacological activation of ALDH2 with AD5591 significantly restored mtDNA content, preserved mitochondrial morphology, improved membrane polarization, and suppressed mitochondrial ROS production, indicating that ALDH2 activity is critical for maintaining mitochondrial homeostasis during ischemia.

**FIGURE 5:**
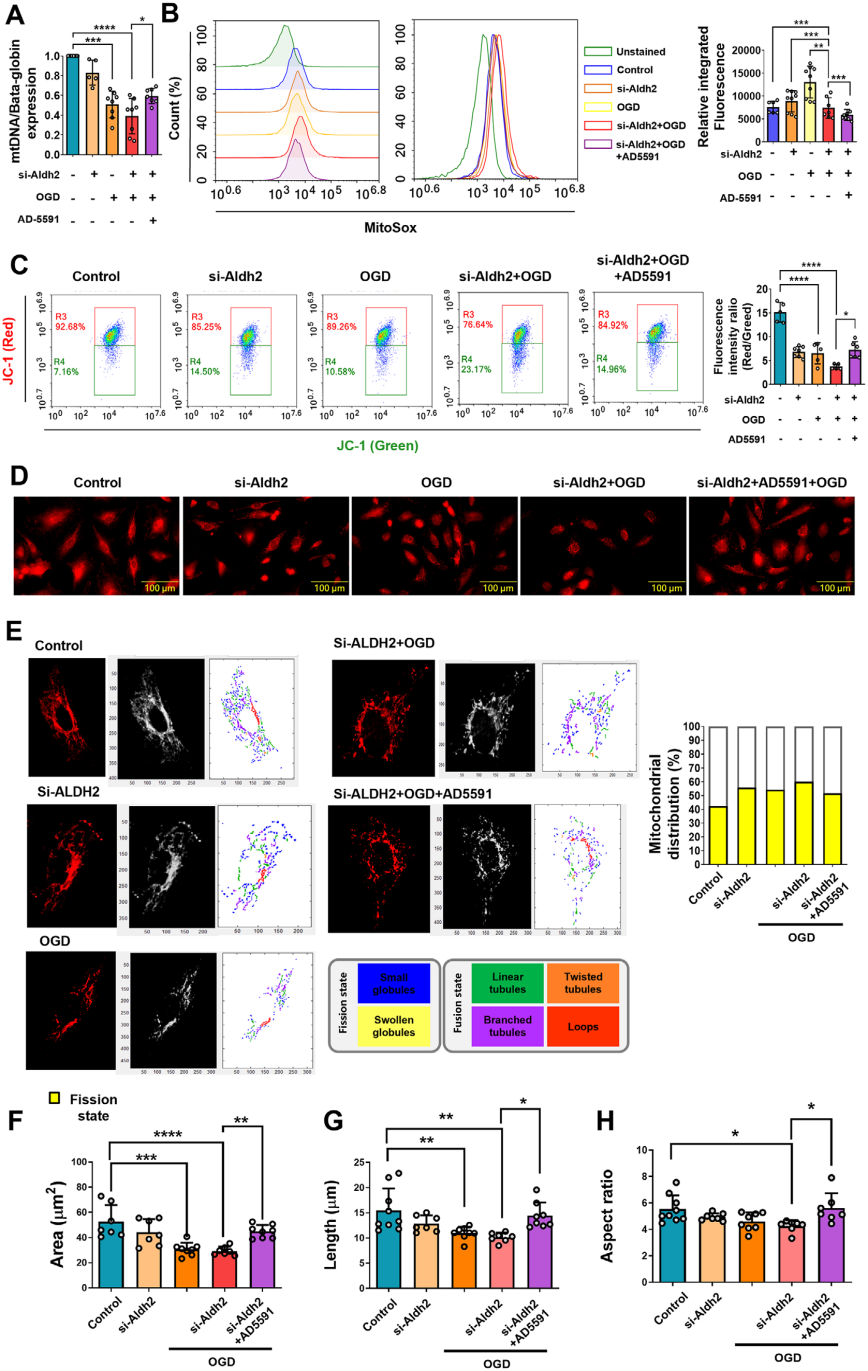
AD5591 mitigated ALDH2 deficiency‐exacerbated mitochondrial dysfunction under ischemic stress. (A) Mitochondrial DNA (mtDNA) damage was evaluated by qPCR of the mtDNA/β‐globin ratio in HUVECs under various conditions: control, siALDH2, OGD, siALDH2 + OGD, and siALDH2 + OGD + AD5591 (ALDH2 activator) (n = 5–8 per group). (B) Flow cytometric assessment of mitochondrial membrane potential using JC‐1 staining demonstrated significant depolarization in siALDH2 + OGD cells, which was restored by AD5591 but reversed by HSP60 knockdown. (C) MitoSOX Red fluorescence indicated excessive mitochondrial ROS generation in siALDH2 + OGD‐treated cells, attenuated by AD5591 and aggravated by HSP60 silencing. (D) Representative confocal images showing mitochondrial morphology by MitoTracker staining. Quantitative analysis of (E) mitochondrial morphology, including fusion/fission states, (F) mitochondrial area, (G) branch length, and (H) aspect ratio, is shown. Quantitative analysis using ImageJ revealed a shift from fused to fragmented mitochondrial networks in ALDH2‐deficient cells. Data are presented as mean ± SEM. Statistical significance was determined using one‐way ANOVA with post hoc testing. ^*^ *p* < 0.05, ^**^ *p* < 0.01, ^***^ *p* < 0.005, ^****^ *p* < 0.001. (N = 6‐8 biologically independent samples).

### 2.6 VDAC‐dependent Mitochondrial Protein Release Contributes to ALDH2‐related Mitochondrial Injury

To further delineate the mechanism linking ALDH2 deficiency to mitochondrial instability, we examined the role of voltage‐dependent anion channel (VDAC)–mediated mitochondrial protein release. Pharmacological inhibition of VDAC, which limits the translocation of the mitochondrial chaperone HSP60 to the cytosol, significantly attenuated mitochondrial fragmentation, restored mitochondrial membrane potential, and reduced mitochondrial ROS accumulation (Figure 6A–K). Also, native gel analysis of mitochondrial and cytosolic fractions demonstrated that treatments of AD5591 and a VDAC inhibitor were associated with preserved higher‐order HSP60 oligomeric assembly and attenuated HSP60 redistribution induced by ALDH2 deficiency and OGD (Figure 6L,M). Among VDAC isoforms, VDAC1 silencing provided the strongest protection against ALDH2 deficiency‐induced mitochondrial injury, significantly reducing HSP60 cytosolic redistribution and preserving mitochondrial localization under ischemic stress (Figure 7A). Consistently, VDAC1 knockdown attenuated mitochondrial fragmentation and restored mitochondrial network integrity, identifying VDAC1 as a key mediator of mitochondrial destabilization downstream of ALDH2 deficiency (Figure 7B). Collectively, these findings indicate that ALDH2 deficiency exacerbates ischemia‐induced mitochondrial dysfunction, at least in part, through VDAC1‐dependent mitochondrial destabilization and HSP60 mislocalization.

**FIGURE 6:**
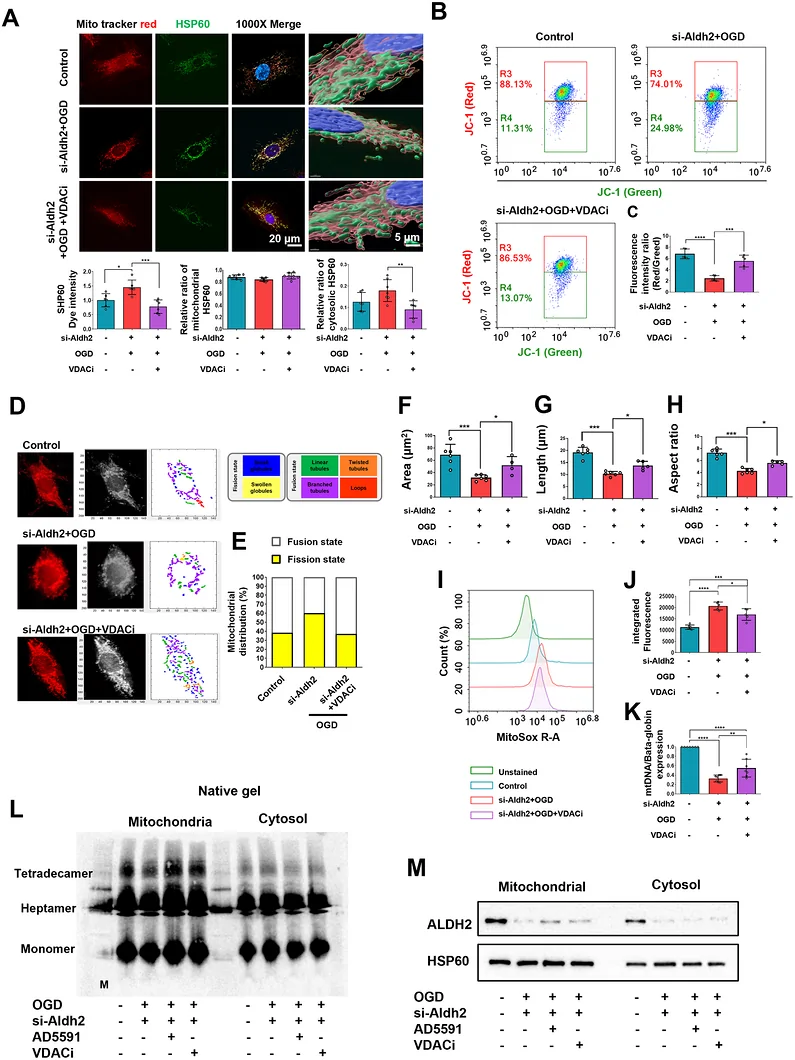
VDAC inhibition attenuates ALDH2 deficiency‐induced mitochondrial dysfunction and preserves mitochondrial HSP60 under ischemic stress. (A) Representative confocal and 3D reconstructed images showing subcellular localization of HSP60 (green) and mitochondria labeled with MitoTracker Red in endothelial cells under control, si‐Aldh2 + OGD, and si‐Aldh2 + OGD + VDAC inhibitor (VDACi) conditions. Quantitative analysis demonstrates changes in total HSP60 intensity, mitochondrial localization, and cytosolic redistribution of HSP60 (n = 5–8/group). (B–C) Representative JC‐1 flow cytometry and quantitative analysis of mitochondrial membrane potential. VDAC inhibition partially restored mitochondrial polarization in ALDH2‐deficient cells exposed to OGD. (D) Representative mitochondrial morphology analysis using the MiNA toolbox. Quantitative analysis of (E) mitochondrial morphology, including fusion/fission states, (F) mitochondrial area, (G) branch length, and (H) aspect ratio, is shown. (I–K) Measurement of mitochondrial reactive oxygen species using MitoSOX, demonstrating increased mtROS accumulation under ALDH2 deficiency and ischemic stress, which was partially reduced by VDAC inhibition. (L) Native gel analysis of mitochondrial and cytosolic fractions showing altered HSP60 oligomeric assembly states (tetradecamer, heptamer, and monomer) under OGD and ALDH2 deficiency. Treatment with AD5591 or VDAC inhibition partially preserved higher‐order HSP60 complex assembly. (M) Representative immunoblot analysis of mitochondrial and cytosolic fractions demonstrating redistribution of ALDH2 and HSP60 under OGD and ALDH2 deficiency. Data are presented as mean ± SEM. Statistical significance was determined by one‐way ANOVA followed by post hoc multiple comparison testing: ^*^ *p* < 0.05, ^**^ *p* < 0.01, ^***^ *p* < 0.005, ^****^ *p* < 0.001. (N = 4‐6 biologically independent samples).

**FIGURE 7:**
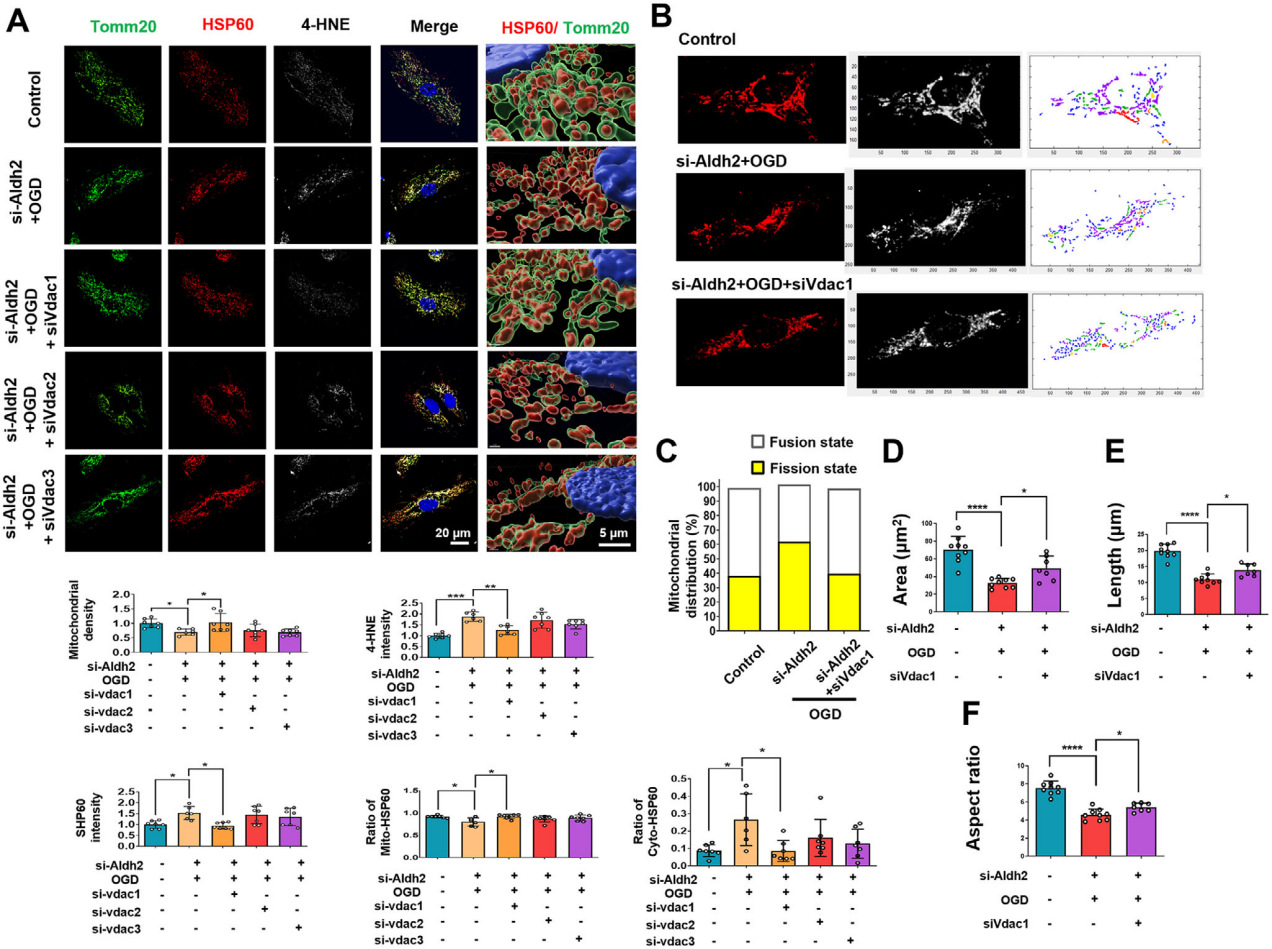
Genetic silencing of VDAC1 preserves mitochondrial integrity and prevents HSP60 redistribution under ALDH2 deficiency and ischemic stress. (A) Representative confocal and three‐dimensional reconstructed images showing the subcellular localization of HSP60 (green) and mitochondria labeled with MitoTracker Red in HUVECs under control, si‐Aldh2 + OGD, si‐Aldh2 + OGD + siVdac1, siVdac2, and siVdac3 conditions. Quantitative analysis of total HSP60 expression, mitochondrial localization, cytosolic redistribution, and mitochondrial fluorescence intensity is shown. (B) Representative MitoTracker images and MiNA (ImageJ) analysis of mitochondrial morphology. VDAC1 silencing attenuated mitochondrial fragmentation and preserved mitochondrial network architecture in ALDH2‐deficient cells exposed to OGD. Quantitative analysis of (C) mitochondrial morphology, including fusion/fission states, (D) mitochondrial area, (E) branch length, and (F) aspect ratio, is shown. Data are presented as mean ± SEM. Statistical significance was determined by one‐way ANOVA followed by post hoc multiple‐comparison testing. ^*^ *p* < 0.05, ^**^ *p* < 0.01, ^***^ *p* < 0.005, ^****^ *p* < 0.001. (N = 4‐6 biologically independent samples).

### 2.7 ALDH2 Polymorphism is Associated with Elevated Oxidative Stress and Increased Risk of MALEs in PAD Patients

A total of 227 patients with PAD were included, comprising 137 without ALDH2 polymorphism (Aldh2 GG) and 90 with ALDH2 polymorphism (Heterozygous: GA = 68, Homozygous: AA = 22). Among them, 70 patients (30.8%) developed major adverse limb events (MALEs) (Table 1). Patients who developed MALEs were significantly older compared with those free from events (73.4±14.6 vs. 69.3±13.8 years, *p* = 0.04) and had a lower proportion of males (54.3% vs. 67.5%, *p* = 0.04). They also exhibited lower body mass index (BMI) (23.2±3.6 vs. 24.9±5.0 kg/m^2^, *p* = 0.01) and a higher prevalence of smoking (54.3% vs. 40.7%, *p* = 0.04). Renal function tended to be worse in the MALE group (eGFR: 37.3±26.9 vs. 45.9±32.4 mL/min/1.73 m^2^, *p* = 0.05). Other comorbidities, including diabetes, hypertension, coronary artery disease, heart failure, hyperlipidemia, prior stroke, and cancer, were comparable between groups. Lipid profiles and liver function tests were also similar. Notably, markers of oxidative stress were significantly elevated in patients with MALEs, including higher circulating levels of 4‐HNE and advanced glycation end products (AGE) (both *p* < 0.01), whereas thiobarbituric acid reactive substances (TBARS) did not differ significantly. Importantly, the prevalence of ALDH2 polymorphism (GA/AA) was markedly higher in the MALE group compared with those without events (90% vs. 17.2%, *p* < 0.001). In univariate analysis, ALDH2 polymorphism was strongly associated with increased risk of MALEs (HR 20.38, 95% CI 9.31–44.63; *p* < 0.001), along with higher 4‐HNE and AGE levels (Table 2). In multivariable models, ALDH2 polymorphism remained an independent predictor of MALEs (Model 1: HR 32.01, 95% CI 13.63–75.14; Model 2: HR 30.41, 95% CI 12.88–71.80; both *p* < 0.001). Smoking and lower BMI were also independently associated with increased risk, whereas AGE levels showed an inverse association after adjustment. Age and sex were not significant predictors. The Kaplan–Meier analysis revealed that PAD patients carrying the ALDH2 polymorphism had a significantly lower probability of remaining free from MALEs compared with those without the polymorphism (*log‐rank p* < 0.01) (Figure 8). This suggests that ALDH2 polymorphism confers an increased risk of limb‐related adverse outcomes, likely mediated through elevated oxidative stress and impaired vascular protection.

**TABLE 1:** The baseline characteristics of patients with peripheral arterial disease (N=227).

Factors	Free from MLAEs (N=157)	MLAEs (N=70)	P value
Age (y/o)	69.3 ± 13.8	73.4±14.6	0.04
Male, n (%)	106 (67.5)	38 (54.3)	0.04
BMI (kg/M^2^)	24.9±5.0	23.2 ± 3.6	0.01
Smoking, n (%)	64 (40.7)	38 (54.3)	0.04
Diabetes, n (%)	95 (60.5)	50 (71.4)	0.13
Hypertension, n (%)	109 (69.4)	47 (67.1)	0.76
CAD, n (%)	69 (43.9)	34 (48.6)	0.56
Heart failure, n (%)	19 (12.1)	10 (14.3)	0.67
Hyperlipidemia, n (%)	69 (43.9)	28 (40)	0.66
Previous stroke, n (%)	24 (15.3)	12 (17.1)	0.69
Cancer, n (%)	10 (6.4)	6 (8.6)	0.58
eGFR(mL/min/1.73m^2^)	45.9 ± 32.4	37.3 ± 26.9	0.05
ALT (mg/dl)	26.4 ± 22.7	22.0 ± 35.5	0.28
Total Cholesterol(mg/dl)	152.4 ± 38.9	156.9 ± 56.1	0.61
LDL (mg/dl)	88.1 ± 34.6	96.4 ± 56.3	0.34
Triglyceride(mg/dl)	162.8 ± 175.7	191.1 ± 332.1	0.55
4HNE(µg/mL)	26.8 ± 25.4	39.0 ± 32.1	0.002
AGE(µg/mL)	29.2 ± 22.9	44.7 ± 24.2	0.001
TBARS(µm)	7.8 ± 4.7	8.6 ± 3.5	0.17
Aldh2 polymorphism (GA or AA)	27 (17.2)	63 (90)	0.001

Data are n (%) or mean ± standard error.; *P <*0.05 as significance.

MALE = major adverse limb events; BMI = body mass index; CAD = coronary artery disease; eGFR = estimated Glomerular filtration rate; ALT = Alanine aminotransferase; LDL = low‐density lipoprotein; 4HNE = 4‐Hydroxynonenal; AGE = advanced glycation end products; TBARS = thiobarbituric acid reactive substances

**TABLE 2:** Univariate and multivariable Cox proportional hazard models of MALEs in patients with PAD.

	Univariate		Multivariable			
			Model 1		Model 2	
	HR (95% CI)	P‐value	HR (95% CI)	P‐value	HR (95% CI)	P‐value
**Age**	1.019 (1.00–1.03)	0.05			1.01 (0.98–1.03)	0.47
**Male gender**	1.54 (0.96–2.47)	0.07			1.21 (0.72–2.03)	0.46
**BMI**	0.94 (0.90–0.98)	0.01	0.92 (0.86–0.98)	0.01	0.92 (0.86–0.99)	0.02
**Smoking**	1.53 (0.95–2.45)	0.07	1.72 (1.05–2.82)	0.03	1.94 (1.13–3.33)	0.01
**eGFR**	0.99 (0.98–1.00)	0.10	0.99 (0.98–1.00)	0.07	0.99 (0.98–1.00)	0.13
**4HNE level**	1.01 (1.01–1.02)	0.001	1.01 (0.99–1.01)	0.06	1.01 (0.99–1.01)	0.07
**AGE level**	1.02 (1.01–1.02)	0.001	0.98 (0.97–0.99)	0.01	0.98 (0.97–0.99)	0.02
**Aldh2 polymorphism**	20.38 (9.31–44.63)	0.001	32.01 (13.63–75.14)	0.001	30.41 (12.88–71.80)	0.001

Abbreviations as listed in Table 1. Model 1 includes smoking, eGFR, 4HNE level, AGE level, and Aldh2 polymorphism. Model 2 includes variables in Model 1 as well as age and sex. Abbreviations are as listed in Table 1.

**FIGURE 8:**
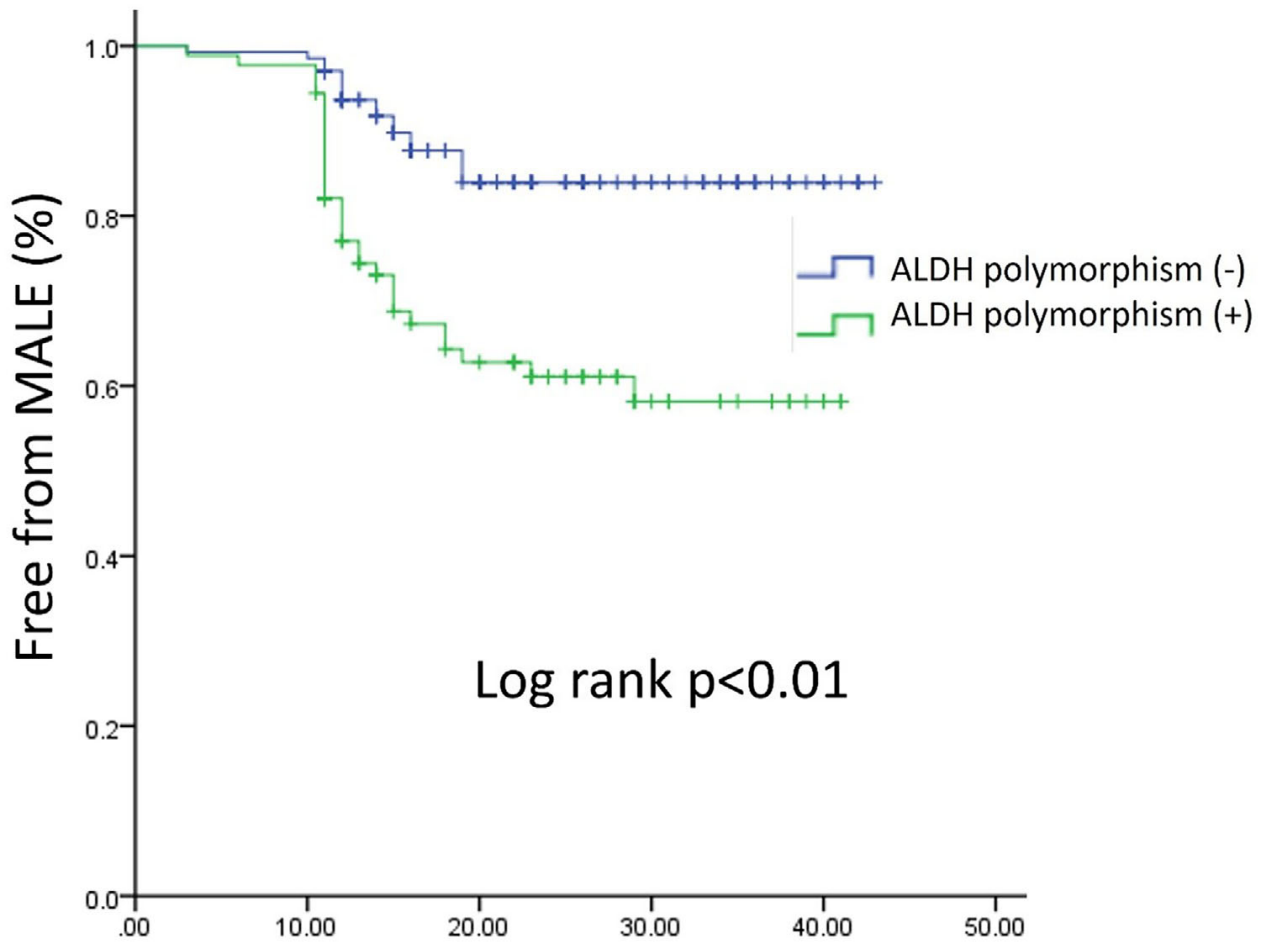
K‐M plot showing that patients carrying ALDH2 rs671 variants (A/A or G/A polymorphism) exhibited higher rates of MALEs than wild‐type carriers in a 227‐patient PAD cohort.

## 3 Discussion

The central observation of this study is that reduced ALDH2 activity couples aldehydic stress to loss of mitochondrial protein homeostasis. Across complementary assays, ALDH2 deficiency increased 4‐HNE, enhanced HSP60 carbonyl modification, altered HSP60 assembly, and favored movement of HSP60 out of the mitochondrial fraction. These events coincided with mtDNA loss, depolarization, mtROS accumulation, network fragmentation, and apoptosis. Rescue by AD‐5591 and by VDAC interference, together with the stronger effect of VDAC1‐specific silencing, supports an ALDH2/4‐HNE‐HSP60‐VDAC1 sequence rather than isolated parallel abnormalities. The association of rs671 with limb events in the clinical cohort provides a translational correlate to the experimental findings (Graphic abstract).

This mechanism extends the conventional view of PAD injury beyond reduced arterial inflow [2]. Ischemic tissue experiences sustained redox pressure, and lipid‐peroxidation products can continue to damage proteins after their initial formation [1, 2, 13]. ALDH2 therefore acts at a biologically important checkpoint by clearing reactive aldehydes [4, 6, 7, 12]. Prior work has linked low ALDH2 activity to impaired endothelial behavior and greater ischemic damage, while ALDH2 restoration is protective [4, 7]. Our data add a protein‐quality‐control component to this framework: when aldehyde clearance is insufficient, HSP60 becomes a target of 4‐HNE and mitochondrial proteostasis deteriorates.

The HSP60 findings are notable because chaperonin function depends on both localization and higher‐order assembly [14, 15]. In our experiments, 4‐HNE modification occurred together with a shift in native HSP60 species and redistribution toward the cytosol. Loss of properly assembled mitochondrial HSP60 provides a mechanistic explanation for the observed deterioration in membrane potential, network morphology, and redox control [8, 14, 15]. Cytosolic HSP60 may additionally participate in stress signaling [9, 15]. Importantly, disrupting VDAC function limited HSP60 redistribution, and VDAC1 silencing was more effective than targeting the other isoforms. We therefore propose that aldehyde‐mediated destabilization of HSP60 proteostasis and VDAC1‐associated redistribution reinforce mitochondrial injury and impair endothelial repair.

Previous studies have commonly used Alda‐1 to activate ALDH2 function [7, 16]. Alda‐1 enhances enzymatic activity by partially blocking the substrate exit tunnel, which facilitates substrate–enzyme interaction without obstructing the catalytic residues Cys302 and Glu268 [7, 17]. However, AD‐5591, a next‐generation ALDH2 activator, demonstrates superior biochemical performance compared with Alda‐1 [7]. Molecular docking analyses revealed that AD‐5591 occupies a similar hydrophobic binding pocket formed by Tyr456, Val458, Lys127, and Phe292, leaving the catalytic site unimpeded. Importantly, unlike Alda‐1, which forms a single hydrogen bond with Asp457, AD‐5591 forms dual hydrogen bonds with both Asp457 and Ala461, resulting in stronger binding affinity and enhanced catalytic stabilization [7]. This dual‐interaction mechanism provides AD‐5591 with greater potency, durability, and selectivity in activating ALDH2 [7]. Consequently, while Alda‐1 laid the foundation for therapeutic ALDH2 activation, AD‐5591 represents a more efficient and stable activator, offering superior mitochondrial protection and clinical potential in oxidative and ischemic vascular injury.

Several constraints should temper interpretation. AD‐5591 rescue was demonstrated primarily in cellular experiments, so therapeutic efficacy still requires direct testing in the in vivo ischemia model [7]. In addition, cultured HUVECs and genetically defined mice simplify the cellular and clinical heterogeneity of human PAD. The proposed HSP60 trafficking step is supported by fractionation, native electrophoresis, co‐immunoprecipitation, PLA, LC‐MS/MS, pharmacologic VDAC inhibition, and isoform‐selective knockdown, but these approaches do not directly image HSP60 crossing the mitochondrial boundary or prove a physical HSP60‐VDAC1 complex. Immunogold electron microscopy, super‐resolution/live‐cell imaging, and dedicated interaction studies could address these points. Finally, because rs671 frequency varies markedly across ancestral populations, the clinical association observed here requires replication in larger and more diverse cohorts before broad generalization. Third, although our findings support a functional ALDH2–4‐HNE–HSP60–VDAC1 signaling axis, we were unable to directly visualize the dynamic transmembrane translocation of HSP60 at the ultrastructural or single‐molecule level or definitively demonstrate a direct physical interaction between HSP60 and VDAC1. Nevertheless, multiple complementary approaches, including subcellular fractionation, native gel electrophoresis, co‐immunoprecipitation, proximity ligation assay, LC–MS/MS, and VDAC isoform‐specific knockdown, collectively support the proposed mechanism of HSP60 redistribution. Future studies employing immunogold electron microscopy, super‐resolution or live‐cell imaging, and advanced protein‐interaction analyses will be important to directly visualize HSP60 translocation and further define its molecular interaction with VDAC1.

## 4 Conclusions

In summary, defective ALDH2‐dependent aldehyde clearance was associated with 4‐HNE modification of HSP60, altered HSP60 proteostasis, VDAC1‐linked redistribution, and mitochondrial injury in ischemic endothelium. Pharmacologic ALDH2 activation and VDAC inhibition each improved key mitochondrial phenotypes, while rs671 carriers in the PAD cohort experienced more adverse limb events. These results nominate mitochondrial HSP60 homeostasis as a mechanistic bridge between ALDH2 deficiency and impaired vascular repair.

## 5 Methods

### 5.1 Study Design of Patients with PAD

Patients with PAD were enrolled prospectively from vascular clinics to test whether rs671 genotype predicted major adverse limb events (MALEs) [7]. The analytic cohort included 227 consented participants after excluding patients unlikely to remain in follow‐up, those scheduled for major amputation, those with active systemic infection, and those expected to survive less than one year. The protocol (IRB 10307‐003; ClinicalTrials protocol CMMC10705‐003) was approved by the institutional ethics committee and conducted in accordance with the Declaration of Helsinki. Baseline clinical characteristics and medications were recorded. MALE was defined as either repeat intervention involving the index arterial segment or amputation of the index limb. Follow‐up lasted a median of 20 months (IQR 12–38), with endpoint status established from visits, chart review, and telephone follow‐up. Genomic DNA from EDTA blood was analyzed for rs671 by PCR‐based microarray and classified as GG, GA, or AA; outcome status was masked from laboratory personnel. Serum 4‐HNE, AGEs, and TBARS were quantified with Cayman ELISA kits.

### 5.2 Animal Study Design and Limb Ischemia Model

C57BL/6 WT and Aldh2 KI mice, 10 weeks of age, were kept on standard chow and water with a 12:12‐h light‐dark cycle. The KI allele contains the previously described G‐to‐A Aldh2 substitution generated by homologous recombination [18]. For hindlimb ischemia, mice received intraperitoneal pentobarbital (80 mg/kg); the left femoral artery and vein were exposed, tied with 6‐0 silk at two points, and the intervening vascular segment removed. The opposite limb underwent exposure only. Experimental assignments comprised WT, KI, WT ischemia, and KI ischemia. Laser Doppler measurements were obtained before surgery and on days 1, 14, and 28, and perfusion was normalized to the contralateral limb [2]. At study completion, animals were euthanized with isoflurane overdose followed by cervical dislocation, and calf muscles were harvested. Both sexes were used. Allocation was randomized, and outcome measurements were performed blinded. Animal procedures were approved by the Chi‐Mei Medical Center animal‐care committee and followed the Guide for the Care and Use of Laboratory Animals.

### 5.3 Histology

Adductor and gastrocnemius samples from both limbs were fixed in paraformaldehyde (4%–10%) or phosphate‐buffered formalin and processed into paraffin blocks. After xylene deparaffinization and graded‐alcohol rehydration, sections underwent heat‐mediated antigen retrieval in pH 6.0 citrate buffer (100°C, 10 min). Nonspecific binding was blocked for 1 h with 5% BSA/PBST. Primary antibodies were applied at 4°C overnight, followed by washing, fluorescent secondary antibodies for 1 h at room temperature, and DAPI nuclear counterstaining.

### 5.4 Capillary Density Analysis and Immunofluorescence

Microvascular density was assessed from CD31‐stained sections using rabbit anti‐CD31 (1:100; Abcam) or Alexa Fluor 594‐conjugated anti‐human CD31 (BioLegend). Olympus BX51 images were collected from five randomly chosen fields per section. ImageJ was used to calculate capillary counts and CD31‐positive area, with field values averaged for analysis. Separate staining for 4‐HNE adducts (1:1000; Abcam) and 8‐OHdG (1:500; Thermo Fisher) was used to evaluate aldehydic/oxidative injury and oxidative DNA damage, respectively.

### 5.5 Cells, ALDH2 Knockdown, AD5591 and VDAC Inhibitor

HUVECs from ATCC were propagated at 37°C/5% CO2 in endothelial medium containing 10% FBS, penicillin (100 U/mL), and streptomycin (50 U/mL). ALDH2 deficiency was modeled with 20 nM siALDH2 delivered by IF‐X2 for 24 h; a non‐targeting sequence served as siRNA control, and knockdown was confirmed by qPCR and immunoblotting. Ischemic stress was simulated by simultaneous exposure to 0.1% O2 and withdrawal of glucose and serum (OGD). When specified, cultures received AD‐5591 (10 µm, 24 h) [7] or VBIT‐12 (10 µm, 24 h) [19].

### 5.6 Pharmacokinetic Analysis of AD‐5591

AD‐5591 pharmacokinetics were determined after one intravenous dose (5 mg/kg) or one oral dose (10 mg/kg) in male C57BL/6 mice. Serial plasma concentrations were measured with a validated assay. Standard noncompartmental calculations provided Tmax, Cmax, t1/2, AUClast, AUCinf, CL, and Vss. Oral bioavailability was derived from dose‐adjusted AUC values assuming linear disposition.

### 5.7 Bromodeoxyuridine (BrdU) Cell Proliferation Assay

For proliferation, 2 × 10^5 HUVECs were placed in each well of a 96‐well plate. After 24 h, BrdU was added, and incubation continued for another 24 h under the assigned oxygen condition. Incorporated BrdU was measured with the Abcam assay at 450 nm using a Multiskan Go reader.

### 5.8 Migration Assay

Migration was measured with Ibidi two‐well inserts. HUVECs (2 × 10^5) were allowed to attach for 24 h, after which the insert was lifted and the monolayer rinsed with PBS. Images obtained immediately and 6 h later under normoxia or 0.1% O2 were analyzed in ImageJ for migration distance. Three independent experiments contributed to the analysis.

### 5.9 Tube Formation Assay

For the angiogenesis assay, Matrigel‐coated 24‐well plates received 2 × 10^5 HUVECs per well. Network formation was photographed with an Olympus BX51 inverted microscope. Total tube length was quantified in ImageJ from five random 100× fields in each well; three independent experiments were evaluated.

### 5.10 Transcriptomic Profiling of ALDH2‐deficient iPSC‐Derived Endothelial Cells

We reprocessed the public bulk RNA‐seq dataset GSE184419, which contains two ALDH2‐corrected isogenic hiPSC‐endothelial lines (Iso_GG) and their uncorrected ALDH2^*^1/^*^2 counterparts [12]. DESeq2 was used for differential‐expression testing with Benjamini‐Hochberg correction (adjusted P<0.05). Significant genes were carried forward to GO and KEGG enrichment, and STRING was used to examine interaction networks relevant to redox, mitochondrial, and endothelial biology.

### 5.11 ALDH2 Enzymatic Activity Assay

ALDH2 activity was quantified from NADH production at 340 nm and 25°C using the previously reported assay [7]. Reactions (100 µL) contained 50 mm sodium pyrophosphate (pH 9.5), 0.01% BSA, 10 mm NAD+, and 50 µM acetaldehyde. Control HUVECs and cells exposed to 5 or 10 µm AD‐5591 were tested, with HepG2 cells included as a positive control. Activity was expressed as µmol NADH generated per minute per milligram of ALDH2 protein.

### 5.12 Mitochondrial Morphology, DNA Copy Number, ROS, and Membrane Potential

MitoTracker Red imaging was used to characterize mitochondrial architecture. ImageJ, together with MicroP and MiNA, provided measurements of footprint, branch length, aspect ratio, and network fusion/fission features [19, 20]. HSP60‐TOM20 colocalization was examined by high‐resolution confocal microscopy and 3D Imaris reconstruction; Pearson coefficients were calculated in ImageJ. Relative mitochondrial DNA content was determined by SYBR qPCR and normalized to nuclear β‐globin. For functional assays, 2 × 10^5 cells/well were exposed for 24 h, then incubated for 30 min at 37°C with MitoSOX Red (5 µm) or JC‐1 (0.1 µm). Fluorescence was measured by NovoCyte flow cytometry after washing.

### 5.13 Mitochondrial Isolation and Subcellular Fractionation

After the assigned OGD/siRNA conditions, cells were fractionated with the Abcam mitochondrial isolation system [21]. Mitochondrial and cytosolic fractions were collected separately, protein concentrations were determined by BCA, and equal amounts were analyzed by SDS‐PAGE/PVDF immunoblotting for ALDH2 and HSP60. GAPDH was used to assess the cytosolic fraction and loading.

### 5.14 Western Blot Analysis

Calf‐muscle homogenates were separated on 10–15% SDS‐polyacrylamide gels and transferred to PVDF. Membranes were probed for ALDH2 and HSP60 (both 1:500; Abcam), VEGF (1:1000; Arigo), cleaved caspase‐3 (1:1000; Cell Signaling), and GAPDH. HRP‐linked secondary antibodies (Jackson ImmunoResearch) and ECL detection (Amersham) were used to visualize immunoreactive bands.

### 5.15 LC–MS/MS Analysis of 4‐HNE‐Modified Proteins

Proteins from mouse tissue were digested with trypsin in solution, desalted with C18 ZipTips, and analyzed by NanoACQUITY UPLC‐Q Exactive HF‐X tandem mass spectrometry. Peptides were resolved with an acetonitrile/formic‐acid gradient and acquired in data‐dependent mode. Mascot 2.6 searched the UniProtKB mouse database using 20‐ppm precursor and 0.02‐Da fragment tolerances and allowing two missed cleavages. Oxidation, deamidation, and 4‐HNE adduction on Cys, His, or Lys were entered as variable modifications; peptide‐spectrum matches were accepted at P<0.05.

### 5.16 Covalent Docking

Covalent docking was performed with SeeSAR [22]. The HSP60 structure (PDB 4PJ1) was prepared in Pipeline Pilot after removal of crystallographic water. Residues implicated by LC‐MS/MS were used to focus candidate covalent sites for 4‐HNE. Docking otherwise used the program defaults, and final geometries and residue contacts were reviewed in PyMOL 3.1.

### 5.17 Native Gel

HSP60 assembly was evaluated under native conditions. Mitochondrial and cytosolic proteins were mixed with nonreducing, SDS‐free loading buffer and were not heat denatured. After gradient native electrophoresis and transfer, HSP60 was detected by immunoblot. Higher‐apparent‐mass bands were interpreted as assembled oligomeric species and lower‐mass bands as dissociated/monomeric HSP60.

### 5.18 Co‐Immunoprecipitation

Cells were lysed on ice in NP‐40 buffer containing protease inhibitors. Equal protein inputs were incubated overnight at 4°C with anti‐HSP60 or matched IgG, followed by capture on protein A/G agarose. Washed immune complexes were eluted and analyzed by SDS‐PAGE/immunoblot for 4‐HNE adducts; input lysates were analyzed alongside the precipitates.

### 5.19 Proximity Ligation Assay (PLA)

HSP60 proximity to 4‐HNE‐modified proteins was measured in fixed cells with a PLA kit (Cell Signaling). Following permeabilization and blocking, samples were incubated with antibodies to HSP60 and 4‐HNE adducts, then with the corresponding PLA probes. Ligation and amplification were completed according to the kit procedure. All groups were imaged using the same acquisition settings; nuclei were counterstained with DAPI, and PLA puncta or signal per cell was quantified in ImageJ.

### 5.20 VDAC Isoform Knockdown

VDAC1, VDAC2, and VDAC3 were knocked down separately with isoform‐selective siRNAs introduced by Lipofectamine 2000. Loss of the targeted protein was confirmed by immunoblot. HSP60 abundance was then compared between mitochondrial and cytosolic fractions to determine which VDAC isoform most strongly affected its redistribution.

### 5.21 Statistical Analysis

Continuous experimental data are presented as mean ± SEM from at least three independent experiments. Multi‐group comparisons used one‐way ANOVA followed by Tukey testing in GraphPad Prism 6.01. In the clinical cohort, time free of MALEs was summarized by Kaplan‐Meier curves and compared by log‐rank testing. Cox proportional‐hazards models were fitted for univariable and adjusted associations with MALEs. Clinical analyses used SPSS 19. All reported tests were two‐sided, and P<0.05 was defined as statistical significance.

## Author Contributions

YCL, NCW, CHC, and WTC conceived the study. YCC, CHC, and WTC performed experiments. YCL, WCW, and WTC analyzed and interpreted the findings. WTC prepared the initial manuscript. WJY provided support related to AD‐5591. CSH, PCH, WJY, YCC, and CHC contributed supervision and funding support. All authors critically reviewed and approved the final manuscript.

## Funding

This study is supported by Chi‐Mei Medical Center and National Science and Technology Council (NSTC 115‐2314‐B‐384 ‐013). Wei‐Ting Chang is also supported by Yushan Fellow Program from Ministry of Education (MOE) in Taiwan.

## Conflicts of Interest

The authors declare no conflicts of interest.

## Supporting information




**Supporting File 1**: advs78668‐sup‐0001‐SuppMat.docx.

**Supporting File 2**: advs78668‐sup‐0002‐TableS1.docx.

**Supporting File 3**: advs78668‐sup‐0003‐SuppMat.tif.

**Supporting File 4**: advs78668‐sup‐0004‐Figure2.tif.

## Data Availability

The data that support the findings of this study are available from the corresponding author upon reasonable request.
