# Supplementary material for: ALDH2 Deficiency Promotes Mitochondrial Oxidative Stress and Adverse Limb Outcomes via VDAC–HSP60 Signaling
Source: Adv Sci (Weinh). 2026 Nov 9:e78668. Online ahead of print. doi: 10.1002/advs.78668 (PMC13748903; doi:10.1002/advs.78668)
Supplement: Supplementary file 1 [file ADVS-9999-e78668-s001.docx]

**Supplemental Table 1.** pharmacokinetic summary of AD-5591 in BL6 mice

| **Pharmacokinetics of AD-5591 in male C57BL6 mice after IV and PO administration** | | | | | | | | | | | |
| --- | --- | --- | --- | --- | --- | --- | --- | --- | --- | --- | --- |
| **Group** | **Compound** | **Route** | **Dosage** | **T_max_** | **C_max_** | **T1/2** | **AUC_last_** | **AUC_INF_** | **CL** | **Vss** | **F*** |
|  |  |  | **(mg/kg)** | **(hr)** | **(ng/mL)** | **(hr)** | **(hr*ng/mL)** | **(hr*ng/mL)** | **(mL/hr/kg)** | **(mL/kg)** | **%** |
| 1 | AD-5591 | IV | 5 | 0.1 | 2860 | 6.01 | 1450 | 1470 | 3410 | 4540 |  |
| 2 | AD-5591 | PO | 10 | 0.5 | 429 | 3.6 | 515 | 516 |  |  | 17.6 |
| *The bioavailability was calculated assuming linear PK. | | | | | | | | | | | |

**Tmax** = Time to reach maximum plasma concentration; **Cmax** = Maximum (peak) plasma concentration;

**T1/2 (t½)** = Elimination half-life; **AUClast** = Area under the concentration–time curve from time 0 to last measurable concentration

**AUC_INF_** = Area under the concentration–time curve from time 0 extrapolated to infinity;

**CL** = Clearance (volume of plasma cleared of drug per unit time); **Vss** = Volume of distribution at steady state
