# Supplementary material for: ALDH2 Deficiency Promotes Mitochondrial Oxidative Stress and Adverse Limb Outcomes via VDAC–HSP60 Signaling
Source: Adv Sci (Weinh). 2026 Nov 9:e78668. Online ahead of print. doi: 10.1002/advs.78668 (PMC13748903; doi:10.1002/advs.78668)
Supplement: Supplementary file 2 [file ADVS-9999-e78668-s002.docx]

**Supplemental Figure legend**

**Supplemental Figure 1.** Volcano plot depicting global transcriptomic changes between ALDH2*1/*2 iPSC-ECs and isogenic corrected controls (Iso_GG). Key oxidative stress–related genes, including GSTP1, HPGD, and HSPD1, are indicated.

**Supplemental Figure 2.** ALDH2 deficiency promotes apoptosis and impairs angiogenic signaling under ischemic stress. (A) Representative and (B) images of western blot of ALDH2, VEGF, caspase-3, and cleaved caspase-3 in the indicated groups. GAPDH served as loading control. Data are presented as mean ± SEM. Statistical significance was determined using one-way ANOVA with post hoc testing; *p < 0.05, **p < 0.01, ***p < 0.005, ****p < 0.001.
